# Potential Salivary Markers for Differential Diagnosis of Crohn’s Disease and Ulcerative Colitis

**DOI:** 10.3390/life11090943

**Published:** 2021-09-09

**Authors:** Kacper Nijakowski, Rafał Rutkowski, Piotr Eder, Marek Simon, Katarzyna Korybalska, Janusz Witowski, Anna Surdacka

**Affiliations:** 1 Department of Conservative Dentistry and Endodontics, Poznan University of Medical Sciences, 60-812 Poznan, Poland; annasurd@ump.edu.pl; 2 Department of Pathophysiology, Poznan University of Medical Sciences, 60-806 Poznan, Poland; rrutkowski@ump.edu.pl (R.R.); msimon@ump.edu.pl (M.S.); koryb@ump.edu.pl (K.K.); jwitow@ump.edu.pl (J.W.); 3 Department of Gastroenterology, Dietetics and Internal Diseases, Poznan University of Medical Sciences, 60-355 Poznan, Poland; piotreder@ump.edu.pl

**Keywords:** inflammatory bowel disease, ulcerative colitis, Crohn’s disease, saliva, biomarkers, differential diagnosis, calprotectin, myeloperoxidase, TNF-α, oral immunity

## Abstract

The properties of the saliva of patients with inflammatory bowel disease (IBD) are poorly recognized. Likewise, the diagnostic potential of saliva for differentiating various forms of IBD is largely unexplored. Therefore, we compared the concentrations of several parameters in unstimulated whole mixed saliva collected in a standardized manner from patients with active IBD unresponsive to conventional therapy. The samples were received from 27 patients with Crohn’s disease (CD), 24 patients with ulcerative colitis (UC), and 51 healthy individuals. Compared to the controls, the salivary concentrations of S100A8/calprotectin, myeloperoxidase, and IgA were significantly decreased in both CD and UC patients. In addition, patients with UC had decreased levels of TNF-R1 and decreased catalase activity. Interestingly, the concentrations of myeloperoxidase and TNF-R1 showed a high differentiation potential for CD and UC (AUC = 0.690 and 0.672, respectively). All these findings are discussed in the context of host defense in the oral cavity, patients’ prior treatment regimens, and smoking habits.

## 1. Introduction

Inflammatory bowel disease (IBD) is a chronic inflammatory disorder of the gastrointestinal tract that affects more than 6 million people worldwide [1]. Its incidence is rising globally, imposing a considerable burden on health services [2]. The etiopathogenesis of IBD is only partly understood and includes both genetic and environmental factors that induce abnormal immune response [3]. Crohn’s disease and ulcerative colitis are the major forms of IBD, which—although different—share many clinical symptoms. The differential diagnosis is based on endoscopic examination with histopathological evaluation of the inflamed tissue [4,5]. Typically, Crohn’s disease localizes to the terminal ileum, while ulcerative colitis occurs most commonly in the rectum and the colon [6]. Moreover, inflammation in Crohn’s disease affects all layers of the intestine, whereas in ulcerative colitis, it is usually limited to the mucosa [7]. Despite these distinctive features, however, in many cases the differential diagnosis can be difficult, which is not facilitated by the lack of specific and reliable disease markers in the blood [8,9].

Examination of the saliva has so far been used mainly for the assessment of oral health. However, its use as a diagnostic medium is rapidly expanding to include systemic conditions. In this respect, saliva can have an enormous range of diagnostic applications in many fields of medicine, including gastroenterology [10]. We have previously uncovered a distinctive pattern of changes in salivary markers in individuals with simple obesity [11,12]. We have also conducted a systematic review to assess whether studies performed to date have identified any salivary biomarkers that can useful in the context of IBD [13]. The most promising biomarkers with the greatest diagnostic potential appeared to be mediators of oxidative stress [14,15,16], certain inflammatory cytokines [17,18,19], and selected miRNAs [20]. It is not clear, however, whether the changes observed were related to a specific form of IBD, or whether they were simply reflective of ongoing inflammation in the gut.

Therefore, in the present study, we aimed to compare salivary concentrations of selected biomarkers in patients with Crohn’s disease and ulcerative colitis to determine whether they could be of predictive value for the differential diagnosis. We chose salivary markers that characterize inflammation, oxidative stress, and host defense. Such findings could facilitate non-invasive diagnostics in inflammatory bowel diseases.

## 2. Materials and Methods

### 2.1. Study Participants

The study group included 51 adult patients of both sexes, with inflammatory bowel diseases, qualified for biological treatment in the Department of Gastroenterology, Dietetics and Internal Medicine, Poznan University of Medical Sciences, between January 2019 and March 2020. Further patient recruitment was prevented by the outbreak of the COVID-19 pandemic. Of the eligible patients, 27 patients were diagnosed with Crohn’s disease and 24 patients with ulcerative colitis. The diagnosis of IBD based on the clinical symptoms and the endoscopic examination, as well as unresponsiveness to previous conventional therapy, was made by specialists in gastroenterology according to standard criteria [4,5,21,22]. Patients with concomitant autoimmune diseases (including diabetes) were excluded.

The control group consisted of 51 healthy individuals presenting for a routine dental examination. These controls were selected to correspond to IBD patients in terms of age, sex, and body mass index (BMI).

### 2.2. Saliva Collection and Analysis

Oral health was examined in all individuals prior to collecting the saliva using routine methods. Patients with periodontal disease or other overt inflammatory lesions in the oral cavity, and patients taking medications known to affect salivation [23], were not included. For this part of the study, saliva samples were collected from patients during qualification for biological treatment.

Unstimulated whole mixed saliva was collected as previously described [24]. Briefly, saliva was collected in the morning at least 2 h after a meal by passive drooling over 20 min. The saliva collected was immediately analyzed for pH and volume, and then centrifuged to remove any debris, aliquoted, and placed at −80 °C until assayed.

For the laboratory, saliva samples were blinded with sequential numbers according to the random selection of patients. All analyses were performed by a single lab technician, and plates were prepared for analysis according to a set schedule.

Salivary concentrations of selected markers were measured with immunoassays and enzymatic colorimetric assays, as per manufacturer instructions and as detailed in Table 1. Total protein salivary concentration was measured with the Bradford method using the Bio-Rad Protein Assay Dye Reagent (Bio-Rad, Munich, Germany).

### 2.3. Statistical Analysis

Since the data did not follow the normal distribution (as determined by the Shapiro Wilk test), medians and quartile ranges were used for descriptive statistics, and the non-parametric Kruskal–Wallis test followed by the Dunn test was used for multiple comparisons. For qualitative variables, the Chi-square test was used. ROC analysis was performed to assess the predictive power in diagnosis for individual markers. The cut-off values were determined according to the Youden index. The significance level for all analyses was set at 0.05. The statistical analysis was performed using Statistica 13.3 (Statsoft, Cracow, Poland) and GraphPrism 9.2.0 (GraphPad Software, San Diego, CA, USA).

## 3. Results

### 3.1. Patient Characteristics

Detailed patient characteristics are given in Table 2.

The pH of saliva from IBD patients tended to be a little lower compared with healthy controls (*p*-value = 0.04 for CD; and *p*-value = 0.05 for UC), but there was no significant difference in this respect between CD and UC patients (Table 3). There was also no significant difference between the groups in the salivary flow.

### 3.2. Selected Salivary Mediators in Patients with IBD

**Figure 1:**
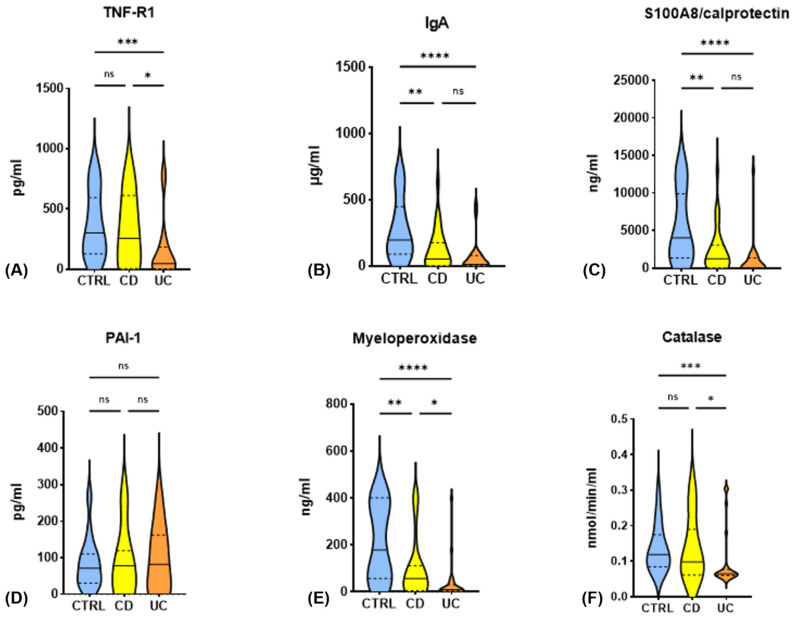
Comparison of salivary concentrations of selected biomarkers between patients with Crohn’s disease and ulcerative colitis (ns—not significant, * *p*-value < 0.05, ** *p*-value < 0.01, *** *p*-value < 0.001, **** *p*-value < 0.0001): (**A**) TNF-R1 (pg/mL); (**B**) IgA (µg/mL); (**C**) S100A8/calprotectin (ng/mL); (**D**) PAI-1 (pg/mL); (**E**) myeloperoxidase (ng/mL); (**F**) catalase (nmol/min/mL).

Compared to the control group, the concentrations of IgA, S100A8/calprotectin, and myeloperoxidase in saliva were significantly lower both in CD patients and in UC patients. In addition, TNF-R1 levels and catalase activity were reduced in UC patients, but not in CD patients. The concentrations of PAI-1 were similar in all groups. Importantly, it was found that patients with UC and CD differed in terms of the concentrations of TNF-R1 and myeloperoxidase, as well as the activity of catalase (Figure 1).

To assess whether these changes were specific or rather reflected the overall changes in salivary protein secretion, the total protein concentration in the saliva was measured and all parameters were normalized per unit of protein.

**Figure 2:**
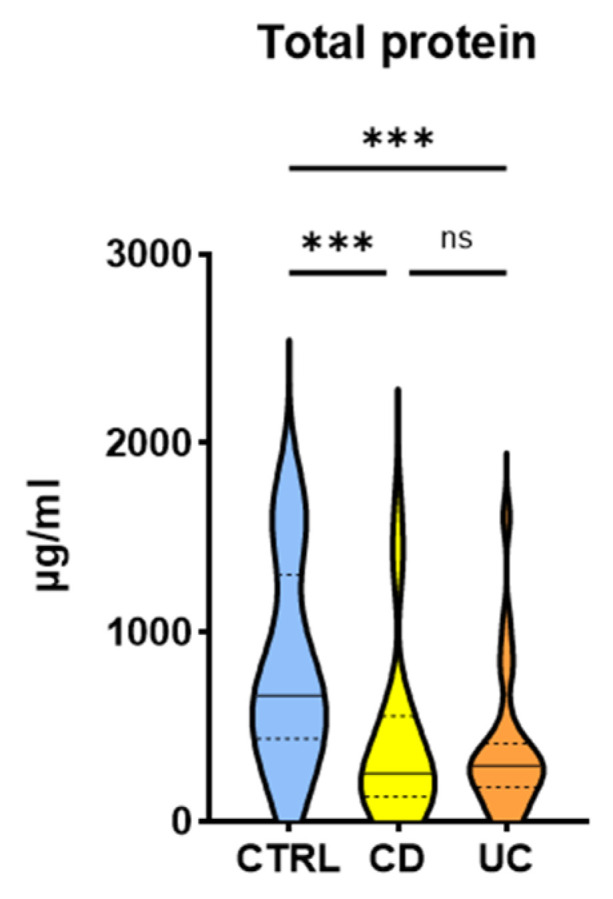
Comparison of salivary concentrations of total protein between patients with Crohn’s disease and ulcerative colitis (ns—not significant, *** *p*-value < 0.001).

The salivary protein concentration was found to be significantly lower (by approximately 60%) in IBD patients compared to the control group (Figure 2). However, there was no difference between concentrations recorded in patients with CD and UC. After normalizing the concentrations of the analyzed parameters in terms of protein content, it turned out that there was still a difference in the levels of TNF-R1 and myeloperoxidase between CD and UC patients (Table 4). Given this consistency, these parameters were selected for further analysis.

### 3.3. ROC Analysis for Predictive Value in Differential Diagnosis between CD and UC

**Figure 3:**
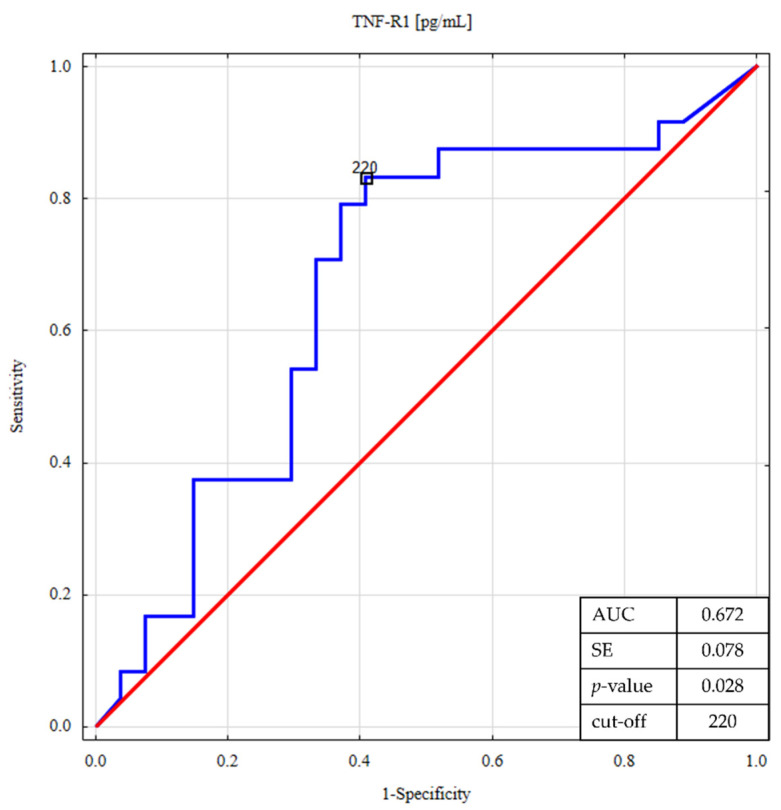
ROC analysis for salivary TNF-R1 in differentiating ulcerative colitis from Crohn’s disease.

**Figure 4:**
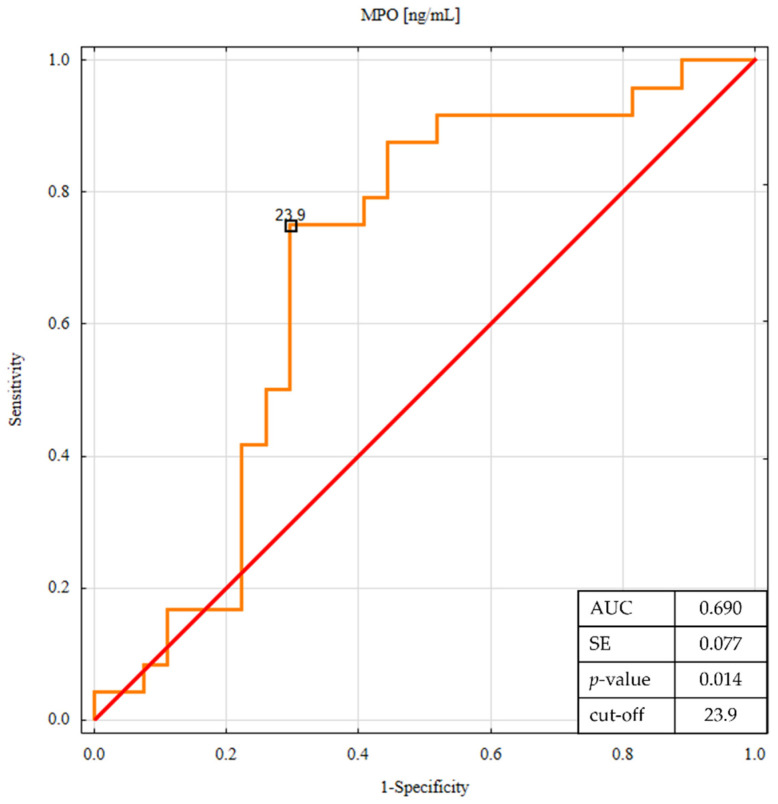
ROC analysis for salivary MPO in differentiating ulcerative colitis from Crohn’s disease.

The ROC analysis was performed to determine whether the salivary concentrations of TNF-R1 and myeloperoxidase discriminate between patients with CD and UC (Figure 3 and Figure 4).

This analysis confirmed that salivary TNF-R1 and MPO levels showed potentially significant predictive values with lower concentrations, which were more likely to be associated with UC. For comparison, Table 5 shows the results of ROC analysis for all salivary biomarkers.

## 4. Discussion

The main finding of the present study is that, contrary to expectations, the salivary concentrations of selected parameters of inflammation, oxidative stress, and host defense can be significantly decreased rather than increased in patients with IBD. Moreover, the magnitude of the decrease in TNF-R1 and MPO levels can be sufficiently different between CD and UC patients to discriminate between these two patient populations.

Of the parameters measured, TNF-R1, myeloperoxidase, catalase, and IgA are well-known mediators of immunity. Therefore, the reduction in their concentrations in saliva may point to impaired host defense in the oral mucosa. Myeloperoxidase in human saliva is largely derived from neutrophils and constitutes a key part of mucosal immunity [25]. It exerts the antimicrobial activity and, together with catalase, protects tissues from oxygen toxicity. Thus, both myeloperoxidase and catalase contribute significantly to the ultimate level of oxidative stress.

In an earlier study, Jahanshahi et al. [16] detected increased oxidative stress in saliva from patients with Crohn’s disease, as evidenced by reduced antioxidant capacity and elevated levels of malondialdehyde (MDA), a product of lipid peroxidation. Since such changes did not occur in patients with ulcerative colitis, the authors suggested that the oral mucosa and salivary glands were not significantly affected by chronic inflammation in UC.

In another cohort of CD patients, Szczeklik et al. [14] found increased levels of salivary MDA, but decreased levels of the reduced form of glutathione and reduced catalase activity. The similar trend for superoxide dismutase was previously noted by the same authors [26]. These authors also demonstrated that compared to CRP, MDA appeared to be more potent in differentiating between active and inactive forms of the disease.

Analyzing the saliva from CD patients, Janšáková et al. [27] observed a reduction in unstimulated salivation, as well as in antioxidant capacity and lactoferrin levels. In contrast, they found no difference in the concentration of myeloperoxidase and detected increased IgA levels.

Szczeklik et al. [17] found an increased salivary concentration of TNF-α in patients with CD and observed that TNF-α levels correlated with the occurrence of specific changes in the oral mucosa. Likewise, Said et al. [18] observed significantly elevated salivary levels of TNF-α in patients with CD. We found that the salivary concentration of TNF-R1, one of the receptors for TNF-α, was significantly reduced in patients with ulcerative colitis relative to patients with Crohn’s disease and the controls.

Owczarek et al. [28] indicated that serum levels of TNF-α receptors, viewed as markers of inflammation, had high sensitivity in assessing the clinical activity of Crohn’s disease and ulcerative colitis. Similar results were obtained by Spoettl et al. [29], who observed significantly higher levels of TNF-R1 in IBD compared to healthy subjects. Increased levels of serum (and possibly salivary) TNF-R1 are the consequence of the receptor shedding from cell surfaces as a self-protective mechanism to neutralize excessive TNF release. Thus, reduced TNF-R1 levels may predispose one to TNFα-mediated inflammatory stress. In addition to a reduction in TNF-R1 and MPO, salivary levels of immunoglobulin A were also found to be significantly reduced in both Crohn’s disease and ulcerative colitis patients relative to the controls.

While this is consistent with the concept of impaired host defense, Warner et al. [30] observed the opposite effect and detected increased secretion of IgA in patients with inflammatory bowel disease. This would correspond to the postulate of Savage et al. [31] that elevated serum IgA levels are associated with inflammation of the mucosal surfaces throughout the gastrointestinal tract, including the oral cavity.

S100A8 protein is a subunit of calprotectin, a well-recognized fecal marker of IBD [32,33]. Thus, the observed reduction in the salivary concentration of S100A8 in patients with IBD can be viewed as surprising. However, S100A8 is an innate immunity effector that can act as an antimicrobial protein [34]. Therefore, the reduction in its salivary concentration would be consistent with a decrease in the host’s oral defense. In contrast, Majster et al. [35] found significantly elevated levels of calprotectin in saliva from patients with Crohn’s disease and ulcerative colitis.

Taken together, a decrease in the concentration of the above parameters in saliva can point to impaired defense mechanisms in the oral cavity rather than an intensity of the inflammatory reaction in the gut. In this respect, it needs to be noted that the concentrations of inflammatory mediators recorded in IBD results not only from disease-associated inflammation, but also from treatment-induced immunosuppression. Interestingly, in the group of our patients, those with ulcerative colitis seemed to receive combined immunosuppression more frequently than those with CD, which could have contributed to lower levels of several mediators seen in these patients. Therefore, if inflammatory mediators were to be assessed in the saliva of IBD patients, it would be critical to optimize the timing of sampling. Ideally, saliva samples should be collected prospectively, starting from the time before any treatment is initiated.

Due to the division of patients into two subgroups based on the form of the disease, a larger sample size would be desirable for further studies to confirm the relationships obtained. However, eligibility of subsequent patients for the study was interrupted by the outbreak of the pandemic. Moreover, it is known that cigarette smoking affects the salivary levels of antioxidants, immunoglobins, and protein [36,37,38,39]. It is also well known, but poorly understood, that cigarette smoking exerts opposite effects in CD and UC [40]. Indeed, our groups of CD and UC patients differed in the proportions of smokers and non-smokers, which might have also contributed to the differences observed.

## 5. Conclusions

Salivary concentrations of several mediators were found to be significantly decreased in patients with IBD. Moreover, TNF-α and MPO concentrations were found to differentiate patients with CD and UC, irrespective of the total protein content in saliva. However, it remains to be determined in larger and sufficiently powered studies whether such differences could be related to prior treatment history and/or smoking habits.

## Figures and Tables

**Table 1:** Assays used to determine the salivary concentrations of selected analytes.

Marker	Manufacturer	Catalog Number
TNF-R1	Bio-Techne, R&D Systems (Minneapolis, MN, USA)	DY225
Serpin E1/PAI-1		DY1786
S100A8/calprotectin		DY8226
Myeloperoxidase		DY3174
IgA	Demeditec Diagnostics (Kiel, Germany)	DEXK276
Catalase	Cayman Chemical Company (Ann Arbor, MI, USA)	707002

**Table 2:** Patient characteristics (M (Q1–Q3)/*n*, %).

Parameter	Control *n* = 51	CD *n* = 27	UC *n* = 24	*p*-Value	*p*-Value CD vs. UC
Sex (female)	17, 33.3	10, 37.0	7, 29.2	0.837	0.767
Age [years]	32 (26–40)	34 (28–48)	32 (24–40.5)	0.302	0.460
BMI [kg/m^2^]	23.39 (21.70–25.00)	22.09 (19.59–24.49)	22.64 (19.67–25.70)	0.199	>0.999
Smokers	-	7, 25.9	1, 4.2	-	0.053
Previous combined immunosupression (steroids + thiopurines)	not applicable	7, 25.9	11, 45.8	-	0.137
Severity of disease (CDAI for CD and Mayo scale for UC)	not applicable	290 (203–332)	9 (8.5–10.5)	-	-
Disease duration [years]	not applicable	8.5 (6–12)	5 (3–10)	-	0.125

**Table 3:** Basic features of saliva and salivation in IBD patients.

Parameter	Control M (Q1–Q3)	CD M (Q1–Q3)	UC M (Q1–Q3)	*p*-Value (Kruskal–Wallis)	*p*-Value CD vs. UC (Dunn)
pH	6.90 (6.80–7.20)	6.84 (6.53–7.01)	6.87 (6.54–7.02)	0.07	0.93
Flow rate [mL/min]	0.25 (0.20–0.57)	0.38 (0.25–0.50)	0.25 (0.25–0.44)	0.33	0.14

**Table 4:** Comparison of concentrations of selected salivary markers (per microgram of total protein) between patients with Crohn’s disease and ulcerative colitis.

Parameter	CD (A)	UC (B)	Control Group (C)	*p*-Value (Kruskal–Wallis)	*p*-Value (Dunn Post-Hoc)		
	M (Q1–Q3)	M (Q1–Q3)	M (Q1–Q3)		A vs. B	A vs. C	B vs. C
TNF-R1 [pg/µg TP]	0.608 (0.264–1.720)	0.199 (0.033–0.526)	0.478 (0.235–1.095)	0.022 *	0.019 ^#^	0.780	0.134
IgA [µg/µg TP]	0.233 (0.001–0.965)	0.047 (0.001–0.188)	0.299 (0.126–0.719)	<0.001 *	0.110	0.249	<0.001 ^#^
calprotectin [ng/µg TP]	4.755 (0.036–9.369)	0.019 (0.004–2.838)	5.786 (2.691–12.498)	<0.001 *	0.007 ^#^	0.584	<0.001 ^#^
PAI-1 [pg/µg TP]	0.185 (0.000–0.899)	0.222 (0.000–0.687)	0.110 (0.044–0.224)	0.602	>0.999	>0.999	>0.999
MPO [ng/µg TP]	0.167 (0.046–0.577)	0.055 (0.011–0.124)	0.239 (0.070–0.685)	<0.001 *	0.015 ^#^	0.975	<0.001 ^#^
catalase [pmol/min/µg TP]	0.542 (0.265–0.908)	0.291 (0.174–0.530)	0.202 (0.102–0.366)	<0.001 *	0.218	<0.001 ^#^	0.141

* *p*-value < 0.05 for the Kruskal-Wallis test; ^#^ *p*-value < 0.05 for the Dunn post-hoc test.

**Table 5:** ROC curve parameters for selected salivary markers in differentiating ulcerative colitis from Crohn’s disease.

Marker	AUC	SE	−95% CI	+95% CI	*p*-Value
TNF-R1 [pg/mL]	0.672	0.078	0.519	0.826	0.028 *
IgA [µg/mL]	0.617	0.081	0.459	0.776	0.147
calprotectin [ng/mL]	0.667	0.077	0.516	0.819	0.030 *
PAI-1 [pg/mL]	0.528	0.083	0.366	0.689	0.736
MPO [ng/mL]	0.690	0.077	0.538	0.841	0.014 *
CAT [nmol/min/mL]	0.671	0.080	0.514	0.829	0.033 *

* *p*-value < 0.05 for the test comparing the ROC curve with the reference line.

## Data Availability

Data available on request from the corresponding author.
